# Development and evaluation of the *IPLAY* program: A protocol for a mixed-methods feasibility study targeting newcomer youth

**DOI:** 10.1371/journal.pone.0284373

**Published:** 2023-04-13

**Authors:** Matthew Y. W. Kwan, Sujane Kandasamy, Jeffrey D. Graham, Jennifer Konopaki, Denver M. Y. Brown

**Affiliations:** 1 Department of Child & Youth Studies, Brock University, St. Catharines, Canada; 2 Department of Family Medicine, McMaster University, Hamilton, Canada; 3 Department of Kinesiology, McMaster University, Hamilton, Canada; 4 Department of Health Research Methods, Evidence & Impact, McMaster University, Hamilton, Canada; 5 WinSport, Calgary, Alberta, Canada; 6 Department of Psychology, University of Texas at San Antonio, San Antonio, TX, United States of America; PLOS: Public Library of Science, UNITED KINGDOM

## Abstract

**Background:**

Physical Literacy (PL) is a synthesis construct that ties together movement competencies with affective, motivational, and knowledge-based elements. It is considered foundational to the development of physical activity-related outcomes. Many diverse organizations and programs have embraced the concept and are implementing programs targeting each of those core elements. However, research has lagged behind its interest and adoption. Among the more prominent gaps is the design and evaluation of programs that aim to increase PL within special populations such as new immigrants or refugee youth.

**Methods:**

The Immigrant-focused Physical Literacy for Youth (*IPLAY*) program is a co-developed evidence-informed 8-week PL program designed for new immigrant and refugee youths who have recently settled in Calgary, Alberta, Canada. This study aims to use a convergent parallel mixed-methods approach to collect, analyse, and interpret quantitative and qualitative data in the evaluation and iteration of the *IPLAY* program.

**Discussion:**

PL programs can be used as a tool to build confidence and physical competencies among newcomer youth. Furthermore, academic-community collaborations in the design and delivery of PL programs can help improve the access and interest for PL programs among newcomer youth. These partnerships are critical and timely considering the recent and upcoming waves of immigration to “arrival cities” across Canada.

## Introduction

Physical inactivity is a major public health concern [1–4]. Regular engagement in physical activity (PA) confers numerous benefits including improved learning [5–8], life skills, and social/emotional development [9–13], academic performance [14, 15], and overall health and well-being [16–19]. Despite the many benefits, less than 40% of Canadian children and youth are meeting the recommended 60 minutes of daily moderate-to-vigorous PA [20, 21]. Importantly, studies have shown that immigrant youth are particularly vulnerable to the risk of physical inactivity compared to non-immigrant peers. A recent study using a nationally representative sample found that youth living in Canada but born elsewhere were significantly less likely to meet daily PA guidelines compared to Canadian-born peers [22].

Canada has one of the highest rates of immigration and acceptance of refugees in the world [23], with the greatest number of newcomers being children and youth [24, 25]. Immigrants represent nearly one-quarter of Canada’s population today, with the highest concentrations living in Ontario (29%), British Columbia (28%), and Alberta (21%), with numbers only projected to rise [26]–including more than 1.2 million immigrants who will be accepted to Canada over the next three years. This has important public health implications as emerging evidence suggests that over time, newcomers to Canada are at an increased risk for poorer health, and greater chronic disease and health burdens compared to those Canadian-born. Although there have been numerous studies that have observed what is commonly referred to as the “healthy immigrant effect”, whereby new immigrants entering Canada are found to be healthier compared to their Canadian-born counterparts [27, 28]; more recent research suggests that this initial health advantage deteriorates over time with settlement [29–31]. Furthermore, there is accumulating evidence that indicates assimilation and acculturation place settled newcomers at greater risk for chronic health conditions including cardiovascular disease, overweight/obesity, and depression when compared to the Canadian-born population [32–35].

Addressing physical inactivity is a complex issue [36], and some experts have argued that youth today lack the necessary fundamental movement skills, motivation, positive feelings toward, and knowledge to be physically active–what is now collectively referred to as physical literacy (PL). PL is a multifaceted concept [37, 38], uniting multiple previously siloed theoretical paradigms, providing a framework for interventions to build upon. Movement competence is identified as a core domain [38, 39], with competencies in movements applied across a wide range of contexts, including on land, air, or on ice [40, 41]. Positive affect, often expressed in terms of fun and enjoyment, and motivational constructs, such as confidence and self-competence, are also central to the concept [42–44]. Lastly, definitions of PL include understanding movement as an essential condition of human experience [42, 43]. At times, the social aspects of movement are considered as an implicit imperative of PL [44]. Individually, each of the core domains of PL are reasonably well-evidenced. For example, the connections between movement skills and perceived competence in relation to participation in PA are well researched [45, 46]. It is hypothesized that developing motor skill competence enhances perceived competence and, in turn, positively affects PA [47]. The model, however, fails to address key psychological components of PL, namely enjoyment, motivation, and knowledge. Following Social Cognitive Theory (SCT) [48], evidence suggests that efforts should be made to help youth successfully learn new motor skills in order to enhance perceived competence (or confidence) and in turn, make PA more enjoyable in order to increase the likelihood of future PA engagement [49].

Developing PL through a wide range of skills, activities, and environments may be particularly beneficial for recent newcomers to Canada [50]. Coming to a new country often demands acculturation into new beliefs, practices, and structures that are foreign [51]. In the case of PA, this might mean learning new activities for which the person has not been exposed, and therefore, lacks the basic competencies for engagement. For example, newcomers immigrating or relocating from warmer tropical, or desert climates may have never been exposed to snow and ice or water for recreation. Learning how to navigate movement within this new context may be a challenge. Consequently, in addition to being limited in terms of participation (e.g., skating for recreation), it poses a health and safety hazard. For instance, slipping on ice could lead to fractures and concussions. As another example, many new community swimming programs are now marketing themselves as ‘learn to swim’ for water safety and drowning prevention. While not exclusive to newcomers, competencies for movement in water could have life or death implications and the transition for youth, may be challenging, as they move into a foreign place, having to learn and experience a new culture while finding new social groups [52].

Overall, the purpose of the current study is to evaluate a newly developed PL-based program targeting new immigrant and refugee youth. In collaboration with a community organization, the current study will co-develop the *I**mmigrant-focused* *P**hysical* *L**iteracy for* *Y**outh (IPLAY)* program and evaluate its impact. Specifically, the research objective of this study will be to examine the feasibility, acceptability, and efficacy of the *IPLAY* program for newcomer youths. We hypothesize that the PL, PA, and overall mental health of newcomer youths will improve from baseline to post-intervention across each 8-week session.

## Materials and methods

### Co-Development of IPLAY program

*IPLAY* will be an 8-week program designed for newcomer youths. Inclusion criteria includes self-identification as a new immigrant and aged 12–16 years. This program will take place at the large community centre of WinSport, in Calgary, Alberta. Youth participants will attend a session once per week for 90 minutes, engaging in novel movement and sport activities aimed at challenging movements on land, air, and ice. This program includes unique games like Gagaball (i.e., an adapted form of dodgeball within an octagon space outdoors), Kin-ball (i.e., a Canadian-developed game utilizing giant inflatable balls), wheelchair basketball, and sledge hockey or ice luge in the arena. The basic idea is that newcomer youths will be able to experience a variety of different movement activities and games within a supportive group-based setting. Trained facilitators with a PL background will lead these sessions specifically targeting participants’ competence (i.e., skill development), confidence (i.e., efficacy to move in different ways), motivation (i.e., interests towards future engagement of activities), and knowledge and understanding (i.e., understanding the benefits of being physically active and understanding how various activities and facilities such as *WinSport* can facilitate their interests). The aim of this study is to co-develop a PL-based program tailored for newcomer youth, implement the program across two separate iterations, and evaluate both sessions using a mixed methods approach.

### Research design

The initial implementation of *IPLAY* will take place in the Fall of 2022 for 8 weeks and then again in the Winter of 2023 for 8 weeks. Each session will include two groups engaging in the same program concurrently, accommodating up to 20 participants in each group. We will use a multipronged recruitment strategy that includes a partnership with local newcomer-focused service organizations to engage the community and enroll eligible participants into the study. First, we will work with these local organizations to plan appropriate strategies and develop promotional materials (including materials translated to different languages as needed). Participation in the IPLAY program will come at no costs to the youth, and those agreeing to take part in the research component will be remunerated $25 in gift cards for each part of the study that they engage in (i.e., one gift card for the baseline assessment of PL, one gift card for the post-program assessment of PL, and one for participation in the qualitative interviews).

The current study will use a mixed-methods approach, with three distinct phases of the evaluation. Firstly, using a pre-post test design, this study will conduct quantitative evaluations of all participants (up to 80 newcomer youths) to examine the impact of the IPLAY program on youth participants’ PL, PA, and its influence on participants’ overall mental health and wellbeing. Secondly, following each 8-week program, qualitative interviews will be conducted with approximately 10 participants in the Fall and Winter sessions, respectively. Together, using the triangulation of the quantitative and qualitative data in addition to process metrics such as program attendance and participation, will help to establish the feasibility, acceptability, and overall impact or efficacy of the program. Written consent from legal guardian and youth participants will be obtained, with all research protocols receiving institutional ethics approval from the Brock University Office of Research Ethics (Project ID # 21–295).

### Quantitative evaluations of IPLAY

All participants will be assessed quantitatively, pre and post program. Each PL domain will be assessed through a combination of direct observation and questionnaires. Specifically, movement competence will be assessed with the PLAYfun tool [53]. The PLAYself tool [53] will be used to assess confidence, motivation, and knowledge and understanding. PA and screen time [54] behaviours as well as mental health and wellbeing [55, 56] will also be assessed using self-reported questionnaires. Mixed effect models will be used to assess whether children’s PL, PA, mental health and wellbeing improve over time and will also test for potential group and sex effects (time by group/sex interactions).

To reflect Bowen et al (2009)’s 8-domain framework for assessing feasibility studies, we will also include the following domains and questions to the quantitative survey package: 1) Acceptability (addressing program satisfaction, barriers/facilitators to participation, preferences of group classes, program sustainability); 2) Implementation (session quality); and 3) Practicality (appropriate time commitment) [57].

### Qualitative evaluations of IPLAY

To gain a much richer and contextually relevant caption of their experiences and potential impact on our outcomes, we will also be conducting a qualitative study of approximately 15–20 participants that enrolled in the *IPLAY* program. According to information power theory, we expect this sample size to provide the context and data necessary to develop robust themes [58]. Specifically, we will be conducting virtual 1:1 semi-structured interviews with youth participants. Interviews will be conducted in English or in the first language requested by participants. In the case where interpretation is required, translations will be conducted in real-time to ensure data integrity. A grounded theory approach will be employed to collect interview data [59, 60]. Each interview will take place immediately following the Fall or Winter *IPLAY* sessions, respectively, aim at eliciting self-assessment of how the program may or may not have impacted each domain of PL, how this program impacted their future plans for maintaining regular physical activities, and to also acquire constructive feedback regarding the program itself. The resultant interviews will be immediately transcribed and analyzed using initial coding, focused coding, axial coding, the comparative method, and memo-writing [60, 61]. We will use triangulation, collect rich data, and have thick description and member checks to ensure validation and credibility. We will make a special effort to enroll participants who attended all sessions and those who missed or ceased participation in sessions in order to capture diverse perspectives. Our data management plan includes the export of all audio transcriptions via Zoom, verbatim transcription of de-identified interviews, and the use of NVIVO software and manual coding for data analysis.

### Data integration of quantitative and qualitative data

Following both independent quantitative and qualitative data collection and analyses (completed in parallel), we will integrate findings using a joint display technique to determine youths’ acceptability of the *IPLAY* program and the efficacy of the program as it relates to changes in PL, PA and/or mental health and wellbeing outcomes. Joint displays are visual displays that are used to explicitly integrate qualitative and quantitative data and for this study will be used at the point of data interpretation to report key points of data convergence and divergence [62, 63]. We will first create a table positioning quantitative and qualitative data, discuss where the convergence and divergence lay, and develop a figure to highlight these points (as they relate to program improvement) and suggest opportunities for future directions.

### Data management plan

Participant data will be anonymized for the data analyses. All reported study outcomes will be grouped and disseminated without any identifying information included. For instance, participants will be assigned a specific unique code (i.e., participant ID) which will then be used in all data files, and not linked in any way to their personal information, except for in a master file which will be password-protected and stored securely (electronically). The master file with identifiers will be kept in a separate, private electronic folder with limited access to the principal investigator and designated research team members. This data will be kept for a period of 10 years.

### Safety considerations

Participants’ safety will be prioritized throughout each IPLAY session throughout the 8 weeks of the program and during the physical pre- and post-assessments. The IPLAY instructors will be trained prior to the beginning of each 8 week session by WinSport staff. This will include familiarization with the facility, proper technique and use of the equipment that will be used during the program, and the safety procedures if an injury should happen which includes ensuring that one of the instructors/staff members remains with the participant while another immediate contacts the onsite WinSport safety patrol and first aid team. Trained research staff will administer the physical pre- and post-assessments. While the tasks included in the assessment are commonly performed by youth in physical education and/or other sport and recreation settings (i.e., running back and forth, throwing, kicking, hopping one leg), some physical risks may be present. For instance, participants may become fatigued when performing the tasks and, as such, rests and water will be provided. Participants will also be reminded that they do not have to perform a task if they do not want to and a demonstration of the task will be performed by the researcher at the participants’ request. Similarly, if an injury should happen during these tasks, the researcher will ensure that one of the instructors or staff members remains with the participant while another immediately contacts the onsite WinSport safety patrol and first aid team. Instructors and research staff will also be certified in emergency first aid and CPR.

## Discussion

Overall, the primary purpose of this feasibility study will be to evaluate a co-developed PL-based program targeting new immigrant and refugee youth. Using a multi-staged mixed-methods approach, we aim to assess whether the *IPLAY* program can be an effective intervention program to reach the newcomer youth population and illicit positive changes in both PL and their overall mental health and wellbeing. The nexus of the quantitative and qualitative approaches proposed will be integrated to provide a rigorous and rich contextualized account of *IPLAY*, including opportunities for future iterations of such community-based programs. Following these mixed methods analyses, we will have key learnings that can be applied to implement evidence-informed programming targeting newcomer youths.

The proposed activities, including the development of a new academic-community partnership, represents a critical first step towards developing a PL-based intervention targeting newcomer youth to Canada. This group is at a greater risk of inactivity and disease burdens as their time in Canada increases [22, 29–31] and therefore it is important to determine possible risk factor modifications. PL offers a unique and holistic framework upon which we build competencies, confidence, motivation, and knowledge for how to sustain lifelong physically active lifestyles [37]. This proposed work will be a catalyst for the continued development and evaluation of such multidimensional and multicomponent interventions aimed at improving short-term behavioural and health outcomes of newcomer youth to Canada.
